# Time’s Up

**Published:** 1871-01

**Authors:** 


### The Bistoury.

ELMIRA, N. Y., JAN., 1871.

The BISTOURY is published Quarterly, upon the 1st of April, July, October and January, at Fifty Cents a year in advance.

### TIME'S UP.

Our subscribers will please remember that the present is the last number of Volume VI, and that therefore their yearly subscriptions are due. This is the second year of the publication of the BISTOURY in Magazine form, so that those persons who have been receiving our journal regularly without ever having paid anything therefor, are two years in arrears, owing us *one dollar*. It will be remembered that we furnished you the BISTOURY *for four years*, at no cost whatever, so that when we ask you for the small subscription price of fifty cents a year, since its conversion into a magazine, you should not hesitate a moment in paying the amount.

We do not desire to force our journal upon any of the old, *free* subscribers, but we do want them to pay up for the two last years, and then stop the journal if they do not want it any longer. We are constantly receiving new subscribers who are willing to pay in advance, and we do not care to enlarge our edition simply for the gratification of chronic "dead heads." Pay up!

